# Anatomic Features of C-shaped Mandibular Second Molars in a Selected Iranian Population Using CBCT 

**DOI:** 10.22037/iej.v13i1.17286

**Published:** 2018

**Authors:** Maryam Janani, Saeed Rahimi, Farnaz Jafari, Masoomeh Johari, Shabnam Nikniaz, Negin Ghasemi

**Affiliations:** a *Endodontics Department, Dental School, Tabriz University of Medical Sciences, Tabriz, Iran; *; b * Dental and Periodontal Research Center, Department of Endodontics, Dental School, Tabriz University of Medical Sciences, Tabriz, Iran; *; c * Department of Endodontics, Dental School, Tabriz Branch, Islamic Azad University, Tabriz, Iran; *; d * Department of Oral and Maxillofacial Radiology, Dental School, Tabriz University of Medical Sciences, Tabriz, Iran; *; e * Department of Periodontology, Dental School, Shahid Sadoughi University of Medical Sciences, Yazd, Iran*

**Keywords:** C-shaped Canal, Canal Configuration, Cone-beam Computed Tomography, Mandibular Second Molar

## Abstract

**Introduction:**

The aim of this retrospective study was to analyze the frequency of C-shaped root canal configuration and characterize mandibular root canal morphology using cone-beam computed tomography (CBCT) with 3D images in an Iranian population.

**Methods and Materials:**

This study consisted of retrospective evaluation of CBCT images from 231 adult patients (153 with bilateral second mandibular molars). Two endodontists examined 384 mandibular second molars of a population in Tabriz, Iran to determine the presence of C-shaped canals and their anatomical characteristics. Root canal configurations were categorized at three different levels. Bilateral or unilateral occurrence of C-shaped canals and their relationship to gender, age and tooth position were examined and statistically analyzed using *chi* squared test and Fisher’s exact test in SPSS 17. The significance level was set at 0.05.

**Results:**

Of 384 mandibular second molars examined, 82 (21.4%) molars from 58 patients had a C-shaped root canal configuration. The prevalence of bilateral C-shaped canals was 15.6% amongst 153 patients with bilateral mandibular second molars. There were no significant differences in the distribution of C-shaped canals with respect to gender or age (*P*=0.06 and *P*=0.86, respectively). Only 4 teeth (4.9%) had the same root canal configuration from the orifice to the apex. In the remainder of the teeth, the cross-sectional root canal configuration changed at different levels of the root.

**Conclusion:**

There were significant variations in the number of roots and canal morphology in mandibular second molars, which should be considered during debridement and obturation of the root canal system.

## Introduction

Failure of the adhesion of the Hertwig’s epithelia root sheath of the root to the buccal and lingual root surfaces is the main etiologic factor in the development of C-shaped root configuration [1]. The prevalence of C-shaped root canals is 2.7 to 44.5% of mandibular second molars, depending on the population involved [2-8]. There are ethnic variations in the prevalence of C-shaped root canal configuration. 

Root canal treatment of mandibular second molars with C-shaped root canal configuration is a challenge due to the presence of a narrow isthmus and thin walls [6]. Therefore, knowledge about the usual configuration of the pulp and the probable variations is very important for the success of endodontic treatment [2]. 

**Figure 1:**
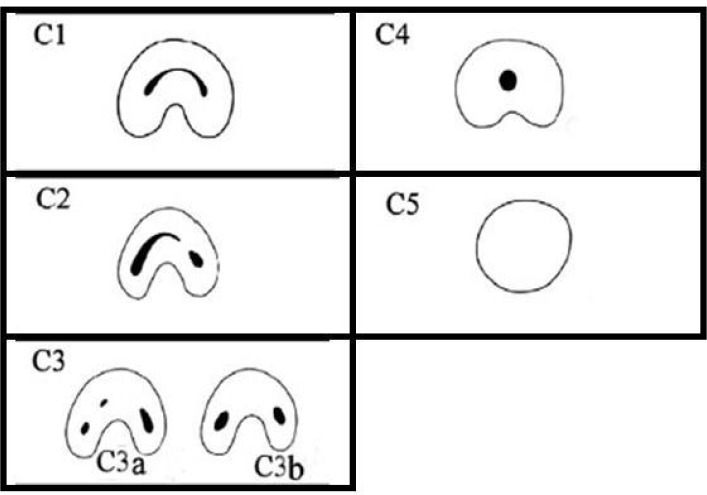
Classification of Fan for C-shaped root canals

A single-rooted mandibular second molar tooth with a continuous orifice for 2, 3 or 4 root canals was reported for the first time in a dental article by Cooke and Cox in 1979 [9]. Menton *et al.* [10] first suggested the classification of C-shaped root canals based on the transverse cross-section shape; however, there was no clear explanation for differentiating groups 2 and 3. Fan *et al.* [3, 4] analyzed the morphology of C-shaped root canals in mandibular second molars with the use of micro-CT and modified Melton technique, as follows (Figure 1): **C1:** one continuous C-shaped root canal with no separation or dividing, **C2:** a comma-shaped root canal, resulting in a non-continuous C-shaped root canal, **C3:** two or three separate root canals, **C4:** only one root canal with a round or oval cross-section and **C5:** absence of any canal cavity, being visible only near the apex [3]. Different techniques have been used to study the internal anatomy of root canals, including conventional and digital radiography of extracted teeth from the mesiodistal and buccolingual aspects, injection of dye into the teeth and cleaning [11, 12], spiral computed tomography scan [13] and micro-computerized tomography [14].

The cone-beam computed tomography (CBCT) technique is a proper, non-invasive and accurate technique with several advantages in epidemiologic endodontic research [1, 7], including a decrease in or elimination of superimposition of adjacent structures and three-dimensional reconstruction (axial, coronal and sagittal) [7], high accuracy, low radiation dose and high scanning speed [1, 7] (it yields images of several teeth with a radiation dose almost similar to that for two periapical radiographs) [15] and a strong relationship between CBCT and histological findings [7]. 

Recently, different studies have been carried out with the use of CBCT to evaluate the prevalence and anatomy of C-shaped root canals [16, 17]. However, only limited studies had been carried out to date in this respect on an Iranian population [18]. Therefore, the present study was undertaken to evaluate the number of roots, the root types, the prevalence of C-shaped root canals in terms of age, gender, bilateral or unilateral nature of this configuration, their classification and changes in the root canal shape of this system along the root.

## Materials and Methods

In the present study, 384 archived CBCT images of mandibular second molars in the Department of Oral and Maxillofacial Radiology, Faculty of Dentistry, Tabriz University of Medical Sciences and one private office were evaluated using simple random sampling technique. All the images had been taken for the treatment needs of the patients. The mandibular second molars included in this study all had fully developed apices and had no periapical lesions, resorption, calcification, open apices, restorations, posts, pervious root canal therapy, extensive restorations, root canal therapy of the adjacent teeth (due to a decrease in the accuracy of evaluations of CBCT technique) and crowns.

A Newtom VGI CBCT unit (NTV; QR SRL Co., Verona, Italy) was used in the present study, with an FOV (field of view) of 3-25 cm and a slice thickness of 0.01 mm, kVp=110, mA=3.5, s=0.01 and mAs=0.01.

The oral and maxillofacial radiologist received proper instructions to be able to use the relevant software program and all the procedural steps were supervised by a radiologist. The axial, coronal and sagittal views were evaluated by two endodontists using the Volume Viewer software program; this software program makes it possible to evaluate the root canals in a 3-dimensional manner. Kappa agreement coefficient was used to evaluate agreement between the observers. When this coefficient was >70% only one of the observers evaluated the rest of the samples; otherwise, a third observer (radiologist) carried out the rest of evaluations.

The C-shaped root canals were evaluated in coronal, middle and apical cross-sections as follows: coronal, 2 mm apical to the orifice; middle, the root length divided by two; apical, 2 mm coronal to the apex. Then these root canals were classified based on the classification suggested by Fan [2, 17].

Data collected at each stage were recorded in specially designed tables and analyzed with SPSS (SPSS version 17.0, SPSS, Chicago, IL, USA). Descriptive data were presented in means (±standard deviations) and qualitative data were presented in frequencies (percentages).

In addition, the unilateral and bilateral occurrence of C-shaped root canals was recorded in patients and its relationship with gender, age and tooth location (left or right side) was determined. Data were analyzed with *chi* squared test and Fisher’s exact test, using SPSS software (SPSS version 17.0, SPSS, Chicago, IL, USA). Statistical significance was defined at *P*<0.05.

**Figure 2:**
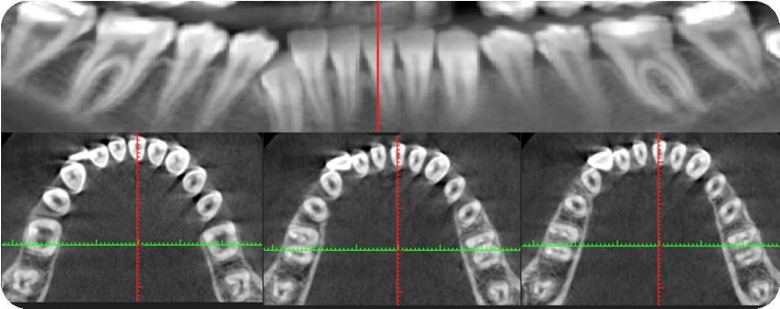
CBCT image and its different cross sections of bilateral mandibular second molars

## Results

Total sample size was calculated at 385 teeth which was estimated by *α*=0.05 and the power of 90% and probable frequency of C-shape anatomy as 0.5. Data collection method was the thorough review of obtained CBCT images with simple random sampling technique. Of 384 mandibular second molars evaluated, 19.8% were single-rooted, 79.2% were two-rooted and 1% were three-rooted, with all the third roots having a distolingual position; 80.7% of two-rooted teeth had three root canals. Based on Vertucci’s classification, the most frequent types of the root canals in two-rooted teeth were type II (52.7%) and type IV (28.3%); in the distal root, 95.7% of the roots were type I based on Vertucci’s classification. 

Evaluation of the CBCT images showed that of 384 mandibular second molars in 231 patients (137 females and 94 males), 82 teeth (21.4%) in 58 patients had C-shaped root canal configuration.

Of these 58 patients, 32 patients had mandibular second molars on both sides and in 24 of them (57.1%), the root canal system was C-shaped on both sides (Figure 2).

**Figure 3:**
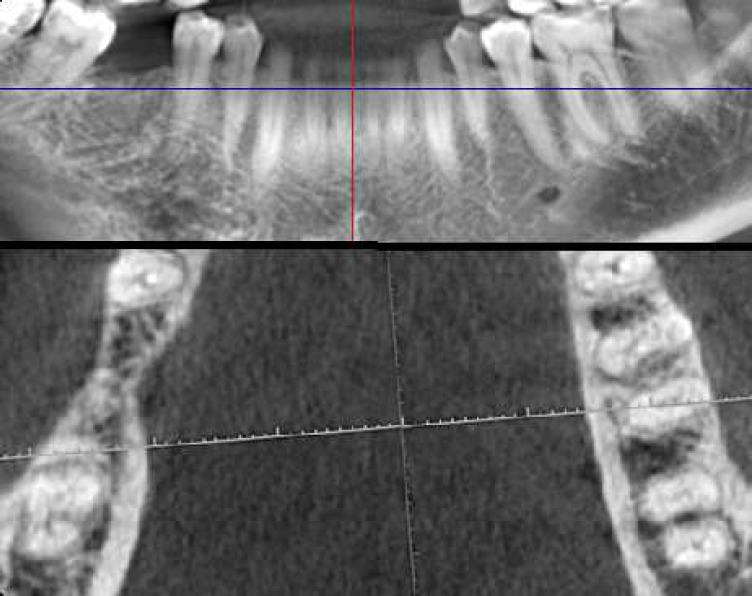
CBCT images of unilateral C-shaped mandibular second molar

Of 153 patients with bilateral mandibular second molars, 24 (15.6%) had C-shaped root canal configuration on both sides and 18 (11.76%) had mandibular C-shaped root canal configuration on unilateral side (Figure 3). 

Of all the C-shaped root canal figurations, 56.25% were on the right side of the mandible, with 43.75% on the left side. However, the difference was not significant (*P*=0.14).

The age range of the subjects was 15‒65 years. The C-shaped root canal configuration was more prevalent in females with 29.9%, compared to 18% in males. However, there were no significant relationships between the frequency of C-shaped root canal configuration and age (*P*=0.86) and gender (*P*=0.06).

In the present study, variations in the C-shaped root canal configuration from the coronal third to the apical third, too, were evaluated. Only 4 teeth had a uniform configuration from the coronal third to the apical third. The rest of the teeth had different forms of the Fan classification system at different cross-section of the root canal.

The most frequent form of C-shaped root canal configuration was C1 in the coronal third. In the middle third the majority of the roots exhibited C3b configuration. In addition, the apical third of the majority of the roots exhibited C3b configuration.

## Discussion

The anatomy of the root canal system affects the outcome of endodontic treatment; therefore, dentists should be aware of the complex anatomy of the root canal system [19, 20].

Based on the results of the present study, the frequency of C-shaped root canal configuration in a Tabrizi population was 21.4%, which is much higher than that reported by Rahimi *et al.* (7.2%), who used the clearing technique to identify C-shaped root canal configuration [2]. The prevalence of C-shaped mandibular second molars in the present study (21.4%) was more than Madani’s investigation (17.6%) which was also applied CBCT images for the investigation [18]. The discrepancy between the results might be attributed to the sample size, the origin and source of the samples and the techniques used for the identification of C-shaped root canal configuration.

Melton *et al.* [10] showed that C-shaped root canal configuration might exhibit changes in the entire length of the root canal system in relation to the shape and number of the root canals. Fan *et al.* [3, 4] confirmed this; therefore, the morphology of the crown or the canal orifice cannot predict the real configuration of the root canal system. 

Fan *et al*. [3, 4] used micro-CT for the analysis of C-shaped root canal system and modified the classification, reporting that C-shaped configuration should have all the characteristics of fused roots and longitudinal concavity on the buccal or lingual surface of the root canal and one cross-section of the root canal should conform to C1, C2 or C3 configuration.

Therefore, in the present study, the cross-section configuration of the root canal was evaluated at coronal, middle and apical thirds of the root. Although different techniques have been used for the evaluation of the root canal system morphology, including placement of files in the root canals, cutting of the root at different levels, construction of a polyester resin cast from the pulp space, and cleaning and injection of dye into the root canal system [2, 17], use of radiography is an easy, effective and non-invasive technique for revealing the morphology of the root canal system [21], which makes it possible to evaluate the root canal system of non-extracted teeth. 

CBCT is more effective than tomography and CT techniques in treatment planning in dentistry. The most important advantage of CBCT is that it provides different views in several planes by a single rotational scanning to provide volumetric data and the relevant images; in addition, its radiation dose is low and it makes it possible to evaluate the relationship between the prevalence and gender and/or age and to evaluate unilateral or bilateral prevalence.

Although C-shaped root canal configuration has been reported in maxillary molars and lateral incisors and maxillary first molars and premolars [21-24], this anatomic variation is usually seen in mandibular second molars [22, 25].

There are definite ethnic differences in the frequencies of C-shaped root canal system configuration [19]. In the present study, the prevalence and anatomic configuration of C-shaped canals were evaluated in an Iranian population in Tabriz.

In the majority of studies in which the morphology of the root canal system has been evaluated [2, 6, 26] the effect of gender has not been evaluated as an effective parameter. Only one study has evaluated it in an Iranian population because it is an ethnicity-related characteristic [2]. However, in the present study, the subjects were divided in terms of gender, and the prevalence and anatomic variations of C-shaped root canal configuration were evaluated in an Iranian population. 

The highest prevalence of C-shaped root canal configuration has been reported in the Korean population (31‒45%) [6].

In the present study, the prevalence of C-shaped root canal configuration was higher than that in a Brazilian population, as reported by two studies with the use of CBCT technique, *i.e.* 3.5% [27] and 15.3% [28]. The discrepancy between the results might be attributed to ethnic differences. 

In another study with the use of CBCT on a Turkish population, the prevalence of C-shaped root canal configuration on mandibular second molars was 8.9% [17], which is less than that in the present study. In addition, its prevalence was higher than those reported by Weine (2.7%) [29] and Cooke and Cox (8%) [9]. On the other hand, its prevalence was less than those reported by Jin *et al.* (44.6%) [25] and Seo and Park (32.7%) [6]. In the present study, similar to the study by Rahimi *et al.* [2] the highest frequency of C-shaped root canal configuration was seen in mandibular second molars that were single-rooted. Although the prevalence of C-shaped root canal system in the Iranian population is less than that of other populations in other centuries, such as China and Korea [1, 5-7], it is important to be familiar with the characteristics of this anatomic variation.

The results of the present study showed that if a mandibular second molar tooth exhibited a C-shaped root canal configuration, the odds of this anatomic variation on the contra-lateral side was 57.1%, consistent with the results of a study by Helvacioglu *et al.* [17], who used the CBCT technique; however, it was different from the results of a study carried out on a Brazilian population with the use of CBCT, in which its prevalence on only one side was 68.3% [28]. Therefore, if an individual has a C-shaped root canal configuration in the mandibular second molar on one side, the dentist should be aware of the high probability of its occurrence on the other side.

In the present study, the cross-section form was evaluated at coronal, middle and apical thirds in the C-shaped root canals. The results showed that only in 4 teeth (4.9%) the root canal configuration was similar in all the three root canal thirds. All the other teeth exhibited 2 or 3 different forms of Fan’s classification, consistent with the results reported by Seo [6] and Zheng [1]. Therefore, preparation of a deep access cavity and accurate probing with a fine file will result in more accurate identification of C-shaped root canal configuration [30]. In the coronal third, C1 form and in the middle and apical thirds C3b forms were the most frequent forms (Table 1). Since cleaning C1 and C2 root canal forms is more difficult than C3 and C4 forms [1], use of alternative techniques such as the ultrasonic technique might be more effective in cleaning the coronal third of C-shaped root canals. Sert and Bayiril [31] used the clearing technique for evaluation of the relationship between gender and root canal morphology and suggested that the patients’ gender, too, should be taken into account in patient assessment before nonsurgical endodontic treatment. Based on the results of the present study, C-shaped root canal configuration was found in 18% of male and 29.9% of female subjects, with no significant relationship between its prevalence and gender (*P*=0.06), consistent with the results of a study by Helvacioglu *et al.* [17] in Turkey and a study by Zheng [1] in China.

**Table 1:** The frequencies of different forms of C-shaped root canal configuration at different cross-sections of the root

**Root canal shape**	**Root cross-section**		
	**Coronal (%)**	**Middle (%)**	**Apical (%)**
**C1**	41 (50%)	7 (8.5%)	5 (6.1%)
**C2**	24 (29.3%)	25 (30.5%)	1 (7.3%)
**C3a**	2 (2.4%)	19 (23.2%)	22 (26.8%)
**C3b**	12 (14.6%)	27 (32.9%)	30 (36.6%)
**C4**	3 (3.7%)	4 (4.9%)	24 (29.3%)
**C5**	0 (0.0%)	0 (0.0%)	0 (0.0%)

In addition, the results of the present study did not show a significant relationship between age (*P*=0.86) and the left or right position and C-shaped root canal configuration (*P*=0.14).

Since the presence of C-shaped configuration is a completely ethnic variation [1, 32], one of the limitations of the present study was a lack of evaluation of the prevalence and anatomic variations of C-shaped root canals in other regions of Iran. Therefore, the values presented here only reflect its regional prevalence and do not indicate its real prevalence all over Iran. 

In the present study a large number of mandibular second molars were two-rooted (79.2%), consistent with the results of studies by Rahimi *et al.* [2] and Weine *et al.* [29]; 81.25% of the teeth had three root canals, 3.6% had 4 root canals and 2% had C-shaped root canals. In Addition, 1% had 3 roots and the third root had a distolingual position.

Based on Vertucci’s classification, the mesial roots of the second molars were type II in 52.7% of the cases and type IV in 28.3% of the cases and the distal root was type I in the majority of cases.

The prevalence of two root canals in the distal root of mandibular second molars that had two roots was 4.3% (types II, III and IV), consistent with the results reported by Ingle [33] and lower than that reported by Rahimi *et al.* (22.5%) [2] and the difference between these two studies might be attributed to differences in the techniques used. 

Considering significant differences in the prevalence and anatomy of C-shaped root canals of mandibular second molars in different parts of the world, further studies are necessary to determine the prevalence and anatomy of these root canals in different ethnic groups in different parts of the world.

## Conclusion

The results of the present study showed considerable variations in the number of roots and morphology of the root canal system of mandibular second molars, with variations in the anatomy of C-shaped root canals at different cross-sections of the root. Therefore, dentists should not believe that mandibular second molars always have two roots and 3 root canals.

The prevalence of C-shaped canals in the mandibular second molars in an Iranian population in Tabriz was lower than that in other Asian countries such as China and Korea and higher than that in Turkey and Brazil.

C-shaped root canal system exhibited anatomic variations at different cross-sections of the root; therefore, during debridement and obturation of the root canal system, these variations should be taken into account.
